# Oridonin-Loaded Nanoparticles Inhibit Breast Cancer Progression Through Regulation of ROS-Related Nrf2 Signaling Pathway

**DOI:** 10.3389/fbioe.2021.600579

**Published:** 2021-04-07

**Authors:** Yue Zhao, Weiwei Xiao, Wanqing Peng, Qinghua Huang, Kunru Wu, Colin E. Evans, Xinguang Liu, Hua Jin

**Affiliations:** ^1^Guangdong Provincial Key Laboratory of Medical Molecular Diagnostics, The Scientific Research Center of Dongguan, College of Pharmacy, Institute of Clinical Laboratory Medicine, Guangdong Medical University, Dongguan, China; ^2^Faculty of Chinese Medicine, Macau University of Science and Technology, Macau, China; ^3^Biosafety Level-3 Laboratory, Guangdong Provincial Key Laboratory of Tropical Disease Research, School of Public Health, Southern Medical University, Guangzhou, China; ^4^Feinberg School of Medicine, Northwestern University, Chicago, IL, United States

**Keywords:** oridonin, PLGA nanoparticle, antitumor activity, breast cancer, cell apoptosis, ROS, Nrf2/HO-1

## Abstract

Oridonin (ORI) has been shown to inhibit tumor cell growth and proliferation *in vitro*, while its optimum anti-tumor activity *in vivo* is limited due to the poor aqueous solubility and bioavailability. In this study, to improve the bioavailability, we developed a nanoparticle-based drug delivery system to facilitate delivery of ORI to breast tumor. ORI was encapsulated in biodegradable nanoparticles (NPs) based on poly-lactic-co-glycolic acid (PLGA) and polyethylene glycol (PEG) to form ORI NPs (ORI-NPs). The resulting ORI-NPs exhibited a mean particle diameter of 100 nm and displayed an efficient cellular uptake by human breast cancer MCF-7 cells. Compared to free ORI that showed no effects on tumor cell proliferation, the ORI-NPs showed significant cytotoxicity and delayed endothelial cell migration, tube formation and angiogenesis. Pharmacokinetics studies showed that ORI-NPs significantly increased the half-life of ORI in the blood circulation. In the nude mouse xenograft model, ORI-NPs markedly inhibited tumor growth and angiogenesis, while ORI did not show any inhibitory effects on the growth of tumor xenografts. The mechanism experiments showed that the antitumor activity of ORI-NPs against breast cancer might be through ROS related Nrf2/HO-1 signaling pathway. Together, these results demonstrated that ORI-loaded PEG-PLGA NPs enhanced bioactivity and bioavailability *in vivo* over ORI, indicating that ORI-NPs may represent a promisingly effective candidate against breast cancer.

## Introduction

Breast cancer is one of the most common cancers diagnosed among women in the world and also the second leading cause of cancer death among women after lung cancer (DeSantis et al., 2014). Most deaths related to breast cancer are caused by metastases in vital organs. The tumor microenvironment is critical for cancer progression, such as growth, dissemination and metastasis (Bonapace et al., 2014). In the tumor microenvironment, endothelial cells play an important role by interaction with tumor cells or formation of blood vessel. The newly formed blood vessels carry oxygen and nutrients to growing tumors, facilitating progression and metastasis. The role of tumor angiogenesis has been demonstrated in many preclinical models and clinical trials, including breast cancer (Batlle et al., 2019; Faulkner et al., 2019; Lang et al., 2019). Thus, anti-angiogenic agent is another kind of promising drug in addition to commonly used cytotoxic anticancer agents for the treatment of breast cancer.

Oridonin (ORI), an ent-kaurene diterpenoid compound isolated from the leaves of *Rabdosia rubescens*, has been proved to possess antibacterial and anti-inflammation activities (Yao et al., 2020). It has been widely used to treat a variety of diseases, including throat swelling, alzheimer’s disease, inflammation of tonsils (Mir et al., 2020). In recent years, lines of evidence have proved that ORI inhibits growth of tumor cells and induces cell apoptosis in some solid tumor types, such as leukemia, lung cancer, colorectal cancer and so on (Zhang et al., 2020). Oridonin inhibits breast cancer cell migration, invasion and adhesion, as well as tumor angiogenesis, which are mediated by suppressing EMT and the HIF-1α/VEGF signaling pathway (Li et al., 2018). Pervious study provides the treatment of ORI can inhibit breast cancer growth and metastasis through blocking the Notch signaling (Xia et al., 2017).

However, due to its poor aqueous solubility and bioavailability, the clinical applications of ORI are greatly hampered. Thus, it is essential to develop an efficient drug delivery system to improve its bioavailability and pharmaceutical properties.

Nanobiotechnology has shown powerful potential to improve drug delivery in cancer and many of these approaches have been applied to breast cancer (Bahreyni et al., 2020). Recently, polymer nanoparticles (NPs) based on biodegradable and biocompatible components are considered as promising efficient carriers for anticancer drugs. The mechanism of PEG-PLGA NPs uptake by cancer cells is mainly through the passive transport associated with enhanced permeability and retention (EPR) effect of tumors. EPR effect of tumors allows NPs with a size in the 20–200 nm range to accumulate in cancer lesions with an impaired vasculature (Maeda et al., 2013). Thus, drugs loading in/onto PEG-PLGA NPs can be easily uptake by cancer cells through improved biocompatibility and bioavailability (Lorenzoni et al., 2019).

The aim of our study is to identify ORI as a compound with anti-breast cancer ability and to construct a drug delivery system that can deliver ORI to tumor cells and improve its effects. Herein, we synthesized ORI loaded PEG-PLGA nanoparticles (ORI-NPs) and evaluated the effects of ORI-NPs on human breast cancer MCF-7 cells *in vitro* and *in vivo*.

## Experimental Section

### Preparation and Characterization of ORI-Loaded PEG-PLGA Nanoparticles

ORI loaded NPs (ORI-NPs) were prepared using emulsification and evaporation method as previously described with modifications (Hua et al., 2017). Briefly, 20 mg of Oridonin (Sigma) and 60 mg of PEG/PLGA (PEG_5000_-PLGA_28,000_, Sigma) were dissolved in 5 ml of dichloromethane as the oil (O) phase, while 20 ml of PVA (1%, w/w, Sigma) was used as external the water (W) phase. The oil phase was sonicated for 40 s at 100 Watt on ice to form the first emulsification, then it was dropped into W phase and sonicated for another 40 s to form the second emulsification. Finally, the nanoparticles were harvested by centrifuging at 12,000 rpm for 20 min and washed three times with water.

The size distribution and zeta potential of the nanoparticles was determined using a Zetasizer Nano ZS (Malvern Instruments, United Kingdom). The morphology of the as-prepared ORI-NPs was characterized by atomic force microscopy (AFM, Autoprobe CP Research, Veeco). AFM scanning were performed on Dimension 3100 system with a tapping mode.

Characterization of the products was confirmed by ^1^H NMR spectrometer (Bruker AVANCE 400 NMR spectrometer, United States) with DMSO as the solvent and Fourier-transform infrared spectrometer (FTIR, Magna FTIR-750). FTIR spectra were obtained from a neat film cast from the chloroform copolymer solution between KBr tablets.

### Determination of Drug Incorporation Efficiency and Release Kinetics of ORI-NPs

To measure ORI incorporation efficiency of ORI-NPs, 10 mg of lyophilized nanoparticles were dissolved in 1 ml of methanol, and then the amount of ORI in solution was determined by high pressure liquid chromatography (HPLC).

The release ratio of ORI from ORI-NPs was determined by HPLC. Briefly, dialysis bags with a molecular weight cut-off of 1,000 Da containing 10 mg of ORI or ORI-NPs in 10 ml PBS (pH 7.4) were immersed in a water bath at 37 C. At indicated time points, 300 μl of sample was removed and replaced with 300 μl of water. Samples were analyzed for ORI concentration by HPLC at 238 nm.

### *In vivo* Pharmacokinetics

The ORI concentration in blood was determined according to the method as previously described (Yao et al., 2020). The ORI-NPs or free ORI solution were intraperitoneally injected into adult BALB/c nude mice (6 weeks, *n = 5*) at a single dose of 10 mg/kg (ORI concentration), and the ORI solution was prepared by dissolving ORI ethanol/water solvent (1/4, v/v). At the indicated time points, 0.2 ml of blood samples were collected into heparinized tubes *via* the retinal vein, and then, was analyzed by HPLC as described above.

### Cell Culture

Human breast adenocarcinoma cells (MCF-7, ATCC) and human umbilical vein endothelial cells (HUVEC, Lonza) were cultured in DMEM (Gibco) with 10% fetal bovine serum (Gibco) containing 100 μg/ml streptomycin and 100 IU/ml penicillin. Cells were routinely subcultured twice a week and incubated in a humidified incubator (Thermo) containing 5% CO_2_ at 37°C.

### Cellular Uptake and Intracellular Location Determination

To determine the NPs uptake by cells, NPs containing a fluorescent dye (Coumarin6) were prepared using the above procedures, except that 250 μg of Coumarin 6 was loaded into the oil phase. The cellular uptake of NPs was qualitatively detected by fluorescence microscopy and flow cytometry (BD FACS Aria). MCF-7 cells at 2 × 10^5^ cells/ml were incubated with different concentrations of Coumarin-6-loaded NPs (1.25, 2.5, 5, and 10 μg/ml) for various periods (0.5, 1, 1.5, and 2 h), and the fluorescence intensity was assessed by confocal microscopy (Leica TCS SP8) and flow cytometry at 488 nm.

To determine the intracellular location, MCF-7 cells at 2 × 10^5^ cells/ml were incubated with 10 μg/ml Coumarin-6-loaded NPs for various periods (0.5, 1 h), LysoTracker Deep Red (100 nM, Invitrogen) was added at the same time. The fluorescence intensity was assessed by confocal microscopy.

### Cell Viability, Apoptosis, ROS, and Mitochondrial Membrane Potential (ΔΨm) Measurement

MCF-7 cells at 5,000/well were seeded in 96-well plates and treated with ORI-NPs at different concentrations for 24 h. The cell viability was evaluated by MTT (Sigma) assay. To determine the cell apoptosis, ROS production and ΔΨm, MCF-7 cells at 2 × 10^5^ cells/ml were firstly seeded in 6-well plates and treated with 10 μg/ml ORI. NPs and ORI-NPs for 24 h. Also, 2.5 mM of NAC were pre-exposure to cells before treated with compounds, and then the viability was assayed by MTT.

Cells were collected and stained with the Annexin V-FITC/PI (BD Biosciences), 2′,7′–dichlorofluorescin diacetate (DCFH-DA, Sigma) and JC-1 (Thermofisher) according to the manufacturer’s instructions, respectively. The fluorescence intensity was assessed by confocal microscopy (Leica TCS SP8) and flow cytometry (BD FACS Aria) at 488 and 594 nM.

### Western Blot Analysis

Western blot assay was performed as previously described. In brief, cells were lysed in RIPA buffer, and lysates were then collected and incubated on ice for 30 min. After centrifuged, supernatant was transferred into clean tubes and protein concentration were measured using BCA assay. Samples were loaded onto an SDS-polyacrylamide gel. The proteins were then transferred to a nitrocellulose membrane. Membrane was blocked in 5% nonfat milk for 1 h at room temperature. Incubated with Nrf2, HO-1, β-actin and Lamin B antibodies (1:1,000 dilution) overnight at 4°C and secondary HRP-linked antibody (1:2,000 dilution) for 1 h at room temperature. Blots were detected by chemiluminescent substrate following the manufacturer’s suggestions and quantitated using ImageJ.

### Wound Healing Assay

Human umbilical vein endothelial cells were cultured at confluence in 6-well plates to obtain confluence. The monolayer was wounded by scratching and washed twice with PBS to remove the detached cells. Then, the cells were cultured in serum-free DMEM media (Gibco) containing different compounds for 24 h. The images of open wound surface area were obtained with an inverted phase-contrast microscope (Nikon Eclipse TS 100). The distance between the edges of defect was determined using the Image J program.

### Tube Formation Assay

After 24-well plates were coated with 100 μl of Matrigel (Corning, #354262) and incubated at 37°C for 30 min, 5 × 10^4^ HUVEC mixed with 10 μg/ml of NPs were seeded on the plates. At 24 h following incubation, the endothelial cell tube formation was assessed by a phase-contrast microscope (Nikon Eclipse TS 100). The number of tubular formations was quantified by manual counting.

### Transendothelial Cell Migration Assay *in vitro*

Cancer cell migration through endothelial barrier was studied in Boyden chambers (6.5mm diameter, 8.0 μm pore size polycarbonate filter; Corning Costar, United States). 2 × 10^5^ HUVECs was seeded on matrigel-coated microwell and incubated for 48 h to obtain the formation of a tight monolayer. HUVEC were treated with 10 μg/ml blank NPs, ORI or ORI-NPs for 90 min. Thereafter, culture media were changed for fresh media, and cells were incubated for an additional 2.5 h. Thereafter, 1 × 10^5^ MCF-7 cells in 100 μl DMEM medium were seeded onto the HUVEC monolayer and transmigration through the endothelial barrier was stimulated by fetal bovine serum (10%) in the lower compartment. The MCF-7 cells were allowed to transmigrate for 18 h, and then the non-migrated cells on the upper surface were removed using a cotton swab. Cell were fixed in 4% (w/v) paraformaldehyde and permeabilized with 0.1% Triton-X100 in PBS. DAPI counterstains cell nucleus. Quantitation was performed by counting the number of tumor cells that had crossed the endothelium with fluorescent microscope (Olympus). All the results represented as normalized values from three independent experiments.

### Chicken Chorioallantoic Membrane Assay

The effects of ORI-NPs on angiogenesis were investigated *ex vivo* using chick embryo CAM assay. Briefly, the fertilized chicken eggs were incubated at 37°C in an atmosphere of 60% humidity, 10 eggs per group. On the seventh day of incubation, a square window was opened in the shell, and different compounds were adsorbed into filter paper (0.5 cm × 0.5 cm) and then put into the CAM. Then, the window was sealed and eggs were returned to the incubator. After another 48 h, arterious branches in CAM were imaged and counted with a digital camera (Nikon, Japan).

### Analysis of Cellular Adherent Junctions by Immunocytochemistry

After the treatments, HUVEC grown on gelatin-coated coverslips were fixed in 4% (w/v) paraformaldehyde and permeabilized with 0.1% Triton-X100 in PBS. Then the cells were incubated with F-actin or VE-cadherin antibodies (Cell signaling) overnight at 4°C and visualized after sequential incubation with indicated secondary antibodies. Pictures were acquired using a confocal microscope (Leica TCS SP8).

### Mouse Xenograft Model

Xenograft model was generated using by injecting MCF-7 cells (1 × 10^7^) in 50 μl matrigel into the flanks of the BALB/c nude mice at 4∼6 weeks of age (Beijing HFK Bioscience). The tumor-bearing mice were randomly divided to the control and the treatment groups (*n = 5*), respectively. At 10 days after tumor cell inoculation, the mice were daily intraperitoneally administrated with saline, blank NPs, ORI (10 mg/kg), or ORI-NPs (10 mg/kg) for 15 days. Previous study of us also injected the same number of cells to build the xenograft model, there was mice in the control group died on the 15 day, so we stopped the experiment. After that, the animals were sacrificed, and the tumors were removed from all animals and weighed. The tumors were fixed for H&E and CD31 staining.

All experimental protocols and procedures were approved by the animal ethics committee of Guangdong Province, China.

### Statistical Analysis

Experimental data were presented as mean ± SD and analyzed using a one-way analysis of variance (ANOVA) with Bonferroni’s test. *P* values of <0.05 were considered as statistically significant.

## Results

### Preparation and Characterization of ORI-NPs

**FIGURE 1:**
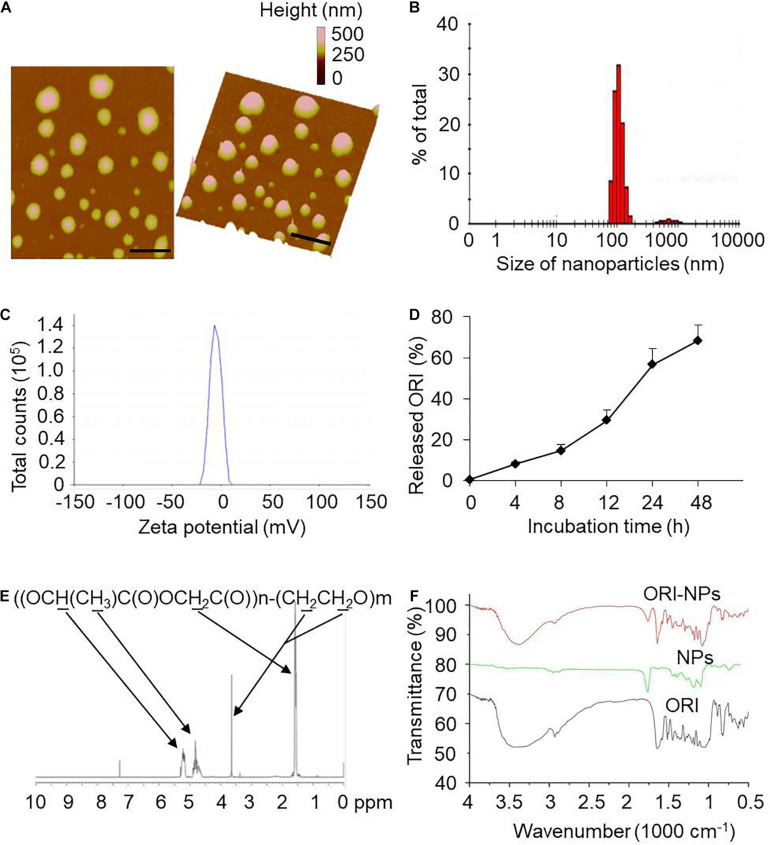
Characterization of ORI-NPs. **(A)** Representative atomic force microscope images of ORI-NPs. **(B)** Size distribution of ORI-NPs. **(C)** The zeta potential distribution analysis of ORI-NPs. **(D)** *In vitro* release kinetics of ORI from ORI-NPs. **(E)** The FTIR spectra of PEG–PLGA NPs, ORI-NPs and ORI. **(F)** 1H NMR spectrum of the synthesized PEG–PLGA–NPs. The copolymer was dissolved in CDCl3 and characterized by 1H NMR at 400 Hz for determining its number-average molecular weight. Characteristic peaks as marked in the graphs.

ORI was encapsulated in PLGA-PEG polymer to form nanoparticles (ORI-NPs) with double emulsion method. The morphology of as-prepared ORI-NPs was characterized by atomic force microscope (AFM). As described in Figures 1A,B, ORI-NPs showed a spherical shape with an average diameter of 100 nm. Zeta potential determination showed that the ORI-NPs were negatively charged with an average value of around −5 mV (Figure 1C).

To determine the *in vitro* release profile of ORI from ORI-NPs, the release kinetics of the as-prepared ORI-NPs were measured by HPLC. As shown in Figure 1D, the release ratio of ORI from ORI-NPs exhibited time dependence, which was significantly increased after 8 h and reached a plateau after 24 h. At 8 and 24 h after incubation in PBS at 37°C, the ratio was 10 and 50%, respectively. Moreover, the incorporation efficiency of drug in ORI-NPs was determined with HPLC assay which showed that the incorporation efficiency of ORI in NPs is about 60%.

Next, the chemical composition of the synthesized product was confirmed by ^1^H HMR as shown in Figure 1E. In consistent with the previous study (Guo et al., 2011), we found that PLGA-PEG NPs showed the characteristic peaks at 1.55, 4.8 and 5.2 ppm belonged to the methyl (d,–CH_3_), methene (m,–CH_2_) and methine (m,–CH) proton of PLGA segment, respectively, and the peak at 3.6 ppm belonged to the methene (s,–CH_2_) proton of PEG chain, confirming the component of PLGA and PEG in the synthesized NPs. As shown in Figure 1F, FTIR spectra further testified the construct of PEG-PLGA NPs. The FTIR spectra of PEG-PLGA NPs and ORI-NPs had similar characteristic peaks.

### Cellular Uptake of ORI-NPs

**FIGURE 2:**
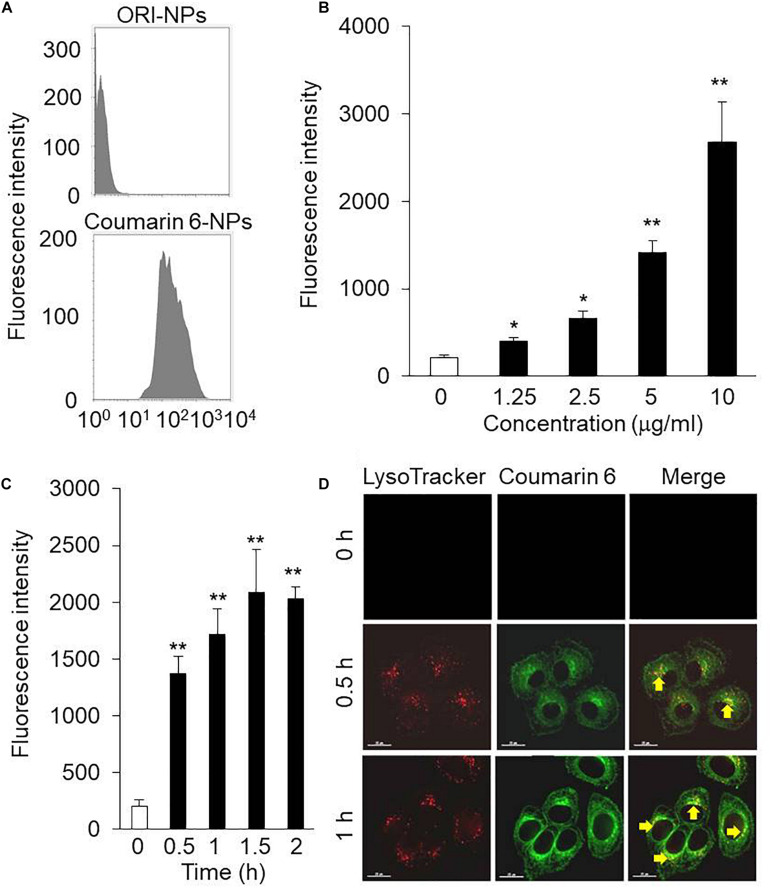
The intracellular uptake of ORI-NPs in MCF-7 cells. **(A)** Flow cytometry analysis showing fluorescence intensity in MCF-7 cells treated with ORI-NPs and Coumarin 6-loaded NPs. **(B)** The dose-dependent cellular fluorescence intensity increased in the MCF-7 cells treated with Coumarin 6-loaded NPs for 2 h were assessed by flow cytometry analysis. **(C)** Time course of cellular fluorescence intensity in the MCF-7 cells treated with 10 μg/ml Coumarin 6-loaded NPs. **(D)** Representative images showing the localization of Coumarin 6-loaded NPs in lysosomes after internalized by the MCF-7 cells, lysosome (red color), Coumarin 6 (green color). Scale bar = 15 μm. (Data were representative three independent experiments. *P < 0.05, **P < 0.01 versus control).

NPs were fluorescently labeled with Coumarin 6, a fluorescent marker, during the synthesis of NPs. For cellular uptake tests, the mean fluorescence intensity (MFI) of the Coumarin 6-loaded NPs in the MCF-7 cells was measured using flow cytometry. As shown in Figure 2A, MFI of MCF-7 cells incubated with ORI-NPs and Coumarin 6-loaded NPs was 3.18 and 318, suggesting that ORI-NPs exhibited weak auto-fluorescence and Coumarin 6 loaded in NPs functioned as a good marker.

Dose and incubation time of nanoparticles are key factors to affect the cellular uptake of nanoparticles. We found that the cellular MFI of NPs in the cells was dependent on the concentration of NPs (Figure 2B). In addition, the MFI significantly increased from 30 min after incubation (Figure 2C).

After uptake of NPs by cells, it is an important step to determine their location in cells, which is associated with their functions and biological effects. As shown in Figure 2D, after 0.5 h incubation, the green fluorescence of Coumarin 6-loaded NPs was observed. In addition, the co-localization of Coumarin 6 loaded NPs and lysosome indicated that the cellular uptake of NPs took place continuously.

### ORI-NPs Induced Cell Growth Inhibition and Apoptosis in Tumor Cells

**FIGURE 3:**
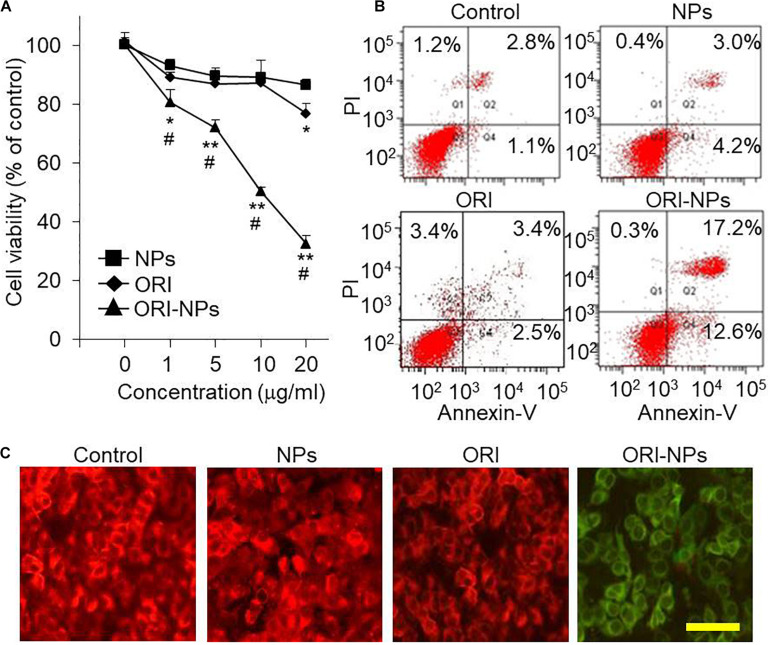
ORI-NPs decreases the cell viability and induced apoptosis of the MCF-7 cells. **(A)** MTT assay demonstrating the inhibitory effects of compounds on the cell viability of MCF-7 cells. After the treatment for 24 h, the cell viability was assessed by MTT assay. **(B)** Cell apoptosis of the MCF-7 cells measured by fluorescence-based flow cytometry analysis. The cells were treated with 10 μg/ml of ORI, blank-NPs or ORI-NPs for 24 h, and then stained with Annexin V/PI for cell apoptosis analysis. **(C)** Representative images of MCF-7 cells stained with JC-1 demonstrating the changes of mitochondrial membrane potential. Scale bar = 20 μm. (Data were representative three independent experiments. *P < 0.05, **P < 0.01 versus blank NPs).

The cellular uptake analysis showed that ORI-NPs could successfully enter into the MCF-7 cells. Therefore, we hypothesize that ORI-NPs exhibit effects on cell growth and proliferation after internalization into cells. Firstly, we determined the effects of free ORI on the cell viability of MCF-7 cells using MTT assay. As shown in Figure 3A, after 24-h treatment, ORI at 10 μg/ml did not show any effects on cell growth and proliferation. By contrast, ORI-NPs at 10 μg/ml showed about 50% inhibition on cell death, while the blank NPs did not show any effects.

For further investigation of the mechanism of cell death induced by ORI-NPs, apoptosis of the MCF-7 cells was assayed using Annexin V/propidium iodide (PI)-based flow cytometry. The results showed a significant late apoptotic cell death of the MCF-7 cells (17.2%) at 24 h after the treatment with ORI-NPs at 10 μg/ml, in contrast with blank-NPs (3%) and ORI (3.4%) (Figure 3B).

### ORI-NPs Induced Disruptions of Mitochondrial Membrane

Mitochondria, the energy factories of cells, are involved in vital processes of cells, such as molecular signal transduction, cell differentiation, cell growth, and cell death. Mitochondria-mediated apoptosis is very important in animal development and tissue homeostasis, and its alteration could lead to malignant disorders including cancer (Szabo et al., 2020).

To monitor mitochondrial function, JC-1, a widely used membrane-permeable fluorescent dye, was employed to evaluate the change of mitochondrial membrane potential (ΔΨm). With the changes of ΔΨm, JC-1 shows different colors from red (high ΔΨm) to green (low ΔΨm). As shown in Figure 3C, ΔΨm in the MCF-7 cells treated with ORI-NPs significantly decreased, suggesting the disruption of mitochondrial membrane in MCF-7 cells.

### ORI-NPs Inhibited the Growth of MCF-7 Through ROS-Related Nrf2 Signal

**FIGURE 4:**
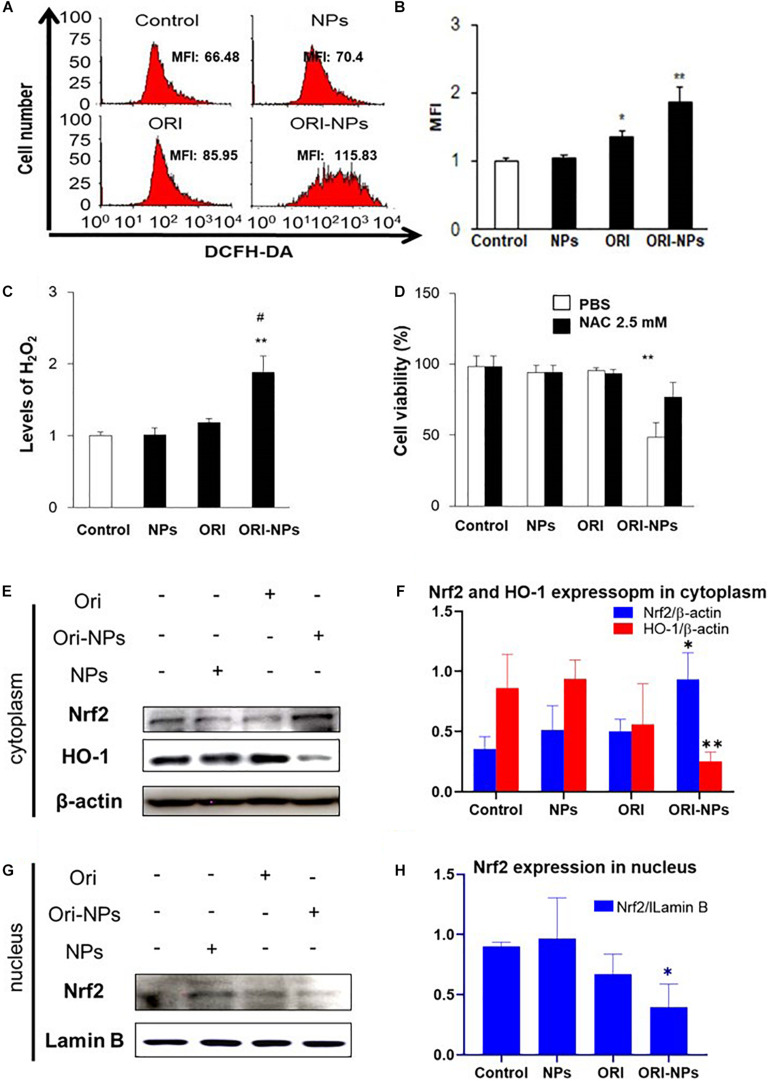
ORI-NPs inhibit MCF-7 cell growth through ROS and Nrf2/HO-1 pathway. **(A,B)** ROS levels in MCF-7 cells treated with drugs for 24 h were measured using flow cytometry. **(C)** H2O2 levels in MCF-7 cells following 24 h treatment with drugs. **(D)** The viability of MCF-7 cells at 24 h treatment with drugs and followed treatment with ROS scavenger, NAC. **(E)** Representative western blot and **(F)** quantification of Nrf2 and HO-1 expression in cytoplasm of MCF-7 cells after 24 h treatment with blank NPs, ORI and ORI-NPs. **(G)** Representative western blot and **(H)** quantification of Nrf2 and HO-1 expression in nucleus of MCF-7 cells after 24 h treatment with blank NPs, ORI and ORI-NPs. β-actin and Lamin B were used as a loading control of proteins in cytoplasm and nucleus, separately. (Data were representative three independent experiments *P < 0.05 and **P < 0.01 versus untreated control; #*P* < 0.05 versus ORI).

Intracellular ROS level plays vital roles in cell apoptotic signaling (Anindya Roy et al., 2020). To determine if ROS was involved in ORI-NPs-induced cell apoptosis, the cellular ROS production of MCF-7 cells was measured by DCFH-DA staining. As shown in Figure 4A, the ROS production in the MCF-7 cells was significantly increased following ORI-NPs treatment (10 μg/ml), but was not significantly changed after the treatment of ORI (10 μg/ml) or blank NPs, implying that elevated ROS production might result from ORI-NPs. The increase of intracellular ROS following ORI-NPs treatment was also confirmed by flow cytometry, which showed a 1.7-fold change in ROS generation (Figures 4B,C). To further confirm the contribution of ROS generation to cell apoptosis, MCF-7 cells were incubated for 4 h with *N*-acetyl-L-cysteine (NAC), a ROS scavenger, prior to the treatment with the agents. NAC significantly reduced the toxicity of ORI-NPs in the MCF-7 cells (Figure 4D), implying the correlation of ORI-NPs-induced cell death with ROS generation. It also showed that ORI-NPs predominantly generated H_2_O_2_ in the MCF-7 cells (Figure 4C).

Evidence showed that Nrf2 pathway could be activated to trigger the expression of antioxidant response element (ARE) target genes heme oxygenase-1 (HO-1), which could attenuate cellular oxidative stress (Wang et al., 2020). To determine the function of ORI on Nrf2 pathway, we examined the nuclear translocation of Nrf2 in MCF-7 cells. Western blot analysis showed that ORI-NP treatment reduced the expression of Nrf2 in the nucleus, contrary to the expression in the cytoplasm. Meanwhile HO-1 expression in cytoplasm also decreased (Figures 4E–H). There were no significant changes in Nrf2 and HO-1 expression in the control, blank-NPs, and free ORI groups. All data indicated that ORI loaded PEG-PLGA NPs can inhibit the Nrf2 nuclear translocation, resulting in a decrease of HO-1 expression. Therefore, the increased level of ROS in cells further promoting cell apoptosis.

### ORI-NPs Inhibited Angiogenesis *in vitro* and *in vivo*

**FIGURE 5:**
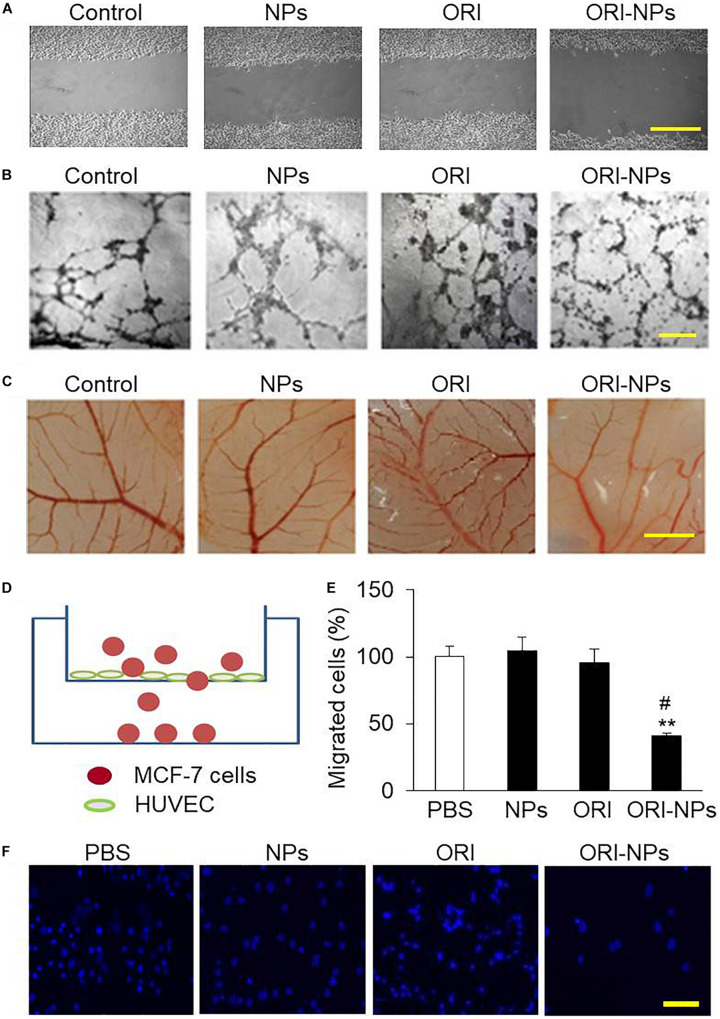
ORI-NPs inhibits angiogenesis and transendothelial migration of MCF-7 cells. **(A)** Wound healing assay demonstrating that ORI-NPs inhibited HUVEC migration. After a total of 1 × 10^5^ HUVEC seeded on 6-well plates obtained confluence, the cell monolayers were treated with PBS, blank NPs, free ORI or ORI-NPs. Then a wound was generated by a scrape with a pipette tip in each well. At 24 h after the scratch, the images of the cell monolayers were captured. Scale bar = 50 μm. **(B)** Representative micrographs of endothelial cell tube formation of HUVEC showing the reduced tube formation by ORI-NPs. Scale bar = 50 μm. **(C)** Representative micrographs of vessel branches in CAM models showing the inhibitory effects of ORI-NPs on angiogenesis. After the incubation for 7 days, the images of CAM were captured. Scale bar = 500 μm. **(D)** Diagram of transwell experiment. **(E)** Representative micrograph showing less migrated MCF-7 cells following ORI-NPs treatment. After the migrated cells in the lower chamber were fixed and stained with DAPI, the photographs were acquired. **(F)** Quantification of number of migrated MCF-7 cells after the treatment with PBS, black NPs, ORI or ORI-NPs. Scale bar = 20 μm. (Data were representative three independent experiments. *P < 0.05 and **P < 0.01 versus PBS; #*P* < 0.05 versus ORI).

Cell migration is critical for endothelial cells to form blood vessels in angiogenesis and thus is necessary for tumor growth and metastasis (Zhou et al., 2014). To determine the effects of ORI-NPs on cell migration, *in vitro* wound healing assay in HUVEC was performed. Representative micrographs of the wound healing at 24 h post-wound were shown in Figure 5A. Application of ORI-NPs (10 μg/ml) markedly delayed the wound closure and healing, whereas the blank NPs or ORI (10 μg/ml) did not show any effects.

The effect of ORI-NPs on tube formation of HUVEC was further investigated on Matrigel layers. After 24 h of incubation, the progress of HUVEC to form tube-like structures was imaged using light microscopy. As shown in Figure 5B, after incubation for 24 h, the HUVEC on Matrigel layer formed a number of tubes. After treated with ORI-NPs (10 μg/ml), the ability of HUVEC to form tubes was significantly reduced.

Angiogenesis is an important step in the progression of tumors. When NPs enter the blood circulation, they are more likely to accumulate in tumor sites which are rich in blood vessels due to the EPR effects of tumors. The chicken chorioallantoic membrane assay (CAM) has been used as an *in vivo* model to assess the effects of anticancer drugs on angiogenesis (Nowak-Sliwinska et al., 2014). The results of CAM assay revealed that angiogenesis of fertilized eggs was clearly observed at 48 h post treatment (Figure 5C). We also found that ORI-NPs at 10 μg/ml remarkably reduced the angiogenesis on the CAM, and the amount of branch vessels, especially small vessels, was significantly decreased. Importantly, ORI-NPs showed stronger inhibitory effects on the blood vessel sprouting compared to ORI. As expected, after the blank-NPs or free ORI treatment, the density and number of microvessels were not significantly decreased.

### ORI-NPs Inhibited Transendothelial Migration

Several lines of evidence have shown that transendothelial migration of tumor cells is a critical step for cellular intravasation from primary loci into vasculature and extravasation into secondary sites, leading to a metastatic colonization (Jean et al., 2014). To gain insights into the effects of ORI-NPs in regulating cell metastasis, we used transwell migration assay (Figure 5D) to determine the migratory response of MCF-7 cells to ORI-NPs. As showed in Figures 5E,F, ORI-NPs significantly inhibited the transendothelial cell migration of MCF-7 cells compared with free ORI, whereas the blank-NPs did not show any effects on the transendothelial migration, implying that the encapsulated ORI but not PLGA-PEG NPs accounted for its bioactivity.

### Pharmacokinetics and Antitumor Activity of ORI-NPs in Mice

**FIGURE 6:**
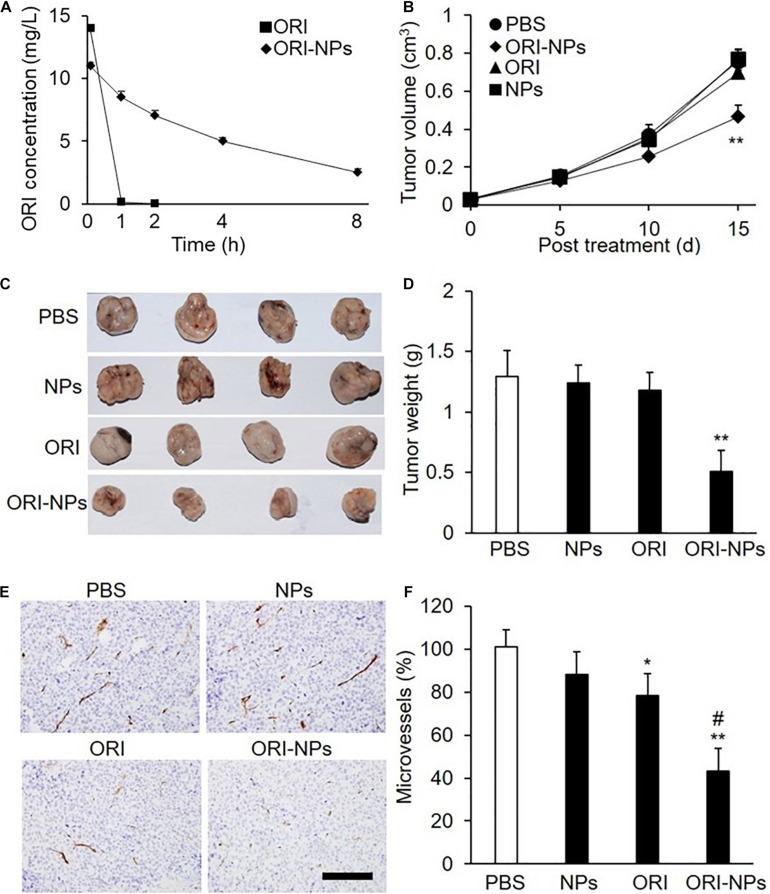
ORI-NPs exhibits prolonged circulation time and inhibitory effects on the growth of the MCF-7 xenografts in nude mice. **(A)** The concentration of oridonin in blood plasma following intraperitoneal administration of free ORI and ORI-NPs in nude mice at the dose of 10 mg/kg for different time. **(B)** Tumor size measured using a caliper and expressed as volume (cm^3^) according to the formula: tumor volume = (length × width^2^)/2. MCF-7 cells (1 × 10^7^) were inoculated subcutaneously into the flanks of nude mice. Ten days later, the mice were intraperitoneal administrated with saline, ORI, blank NPs or ORI-NPs (*n = 5*, 10 mg/kg) once daily for 15 days. **(C)** Representative micrographs of tumors at study end point. **(D)** Quantification of tumor xenograft weights at days 25 after inoculation. **(E)** Representative micrographs of microvessels stained with anti-CD31 antibody. Data representative of five independent experiments. Scale bar = 100 μm. **(F)** Quantification of microvessel numbers in tumor xenografts. (**P < 0.01 versus PBS; #*P* < 0.05 versus ORI).

To determine the half-life of ORI-NPs and free ORI *in vivo*, the plasma concentrations of ORI were determined by HPLC following intraperitoneal injection of ORI-NPs or ORI at 10 mg/kg in mice. Consistent with the previous studies, ORI was rapidly cleared from the systemic circulation (Figure 6A). In contrast, following its encapsulation in NPs, much longer circulation time was achieved. ORI-NPs, in which ORI was conjugated to PEG, demonstrated a half-life of 4 h compared to 0.2 h with free ORI.

As shown above, ORI-NPs exhibited significant inhibitory effects on tumor cells *in vitro*. Further, employing the MCF-7 breast tumor xenograft in nude mice, we determined the ability of ORI-NPs to inhibit tumor cells *in vivo*. The results revealed that ORI-NPs significantly inhibited the growth of the tumor xenografts, as evidenced by the decreased tumor size and weight (Figures 6B–D). Interestingly, ORI disperse in water did not show any inhibition effects.

To assess the ability of ORI-NPs to inhibit angiogenesis *in vivo*, the collected tumor xenografts were sectioned and stained with anti-CD31 antibody to show the vessels. Immunostaining analysis revealed that the application of ORI-NPs markedly reduced the numbers of microvessels in tumor xenografts (Figures 6E,F). Accordingly, blank NPs or ORI did not exert any effects.

## Discussion

As a promising compound with anti-cancer activity against a number of tumors, application of ORI as a therapeutic agent is limited by the poor aqueous solubility and rapid clearance. To prolong its biological half-time, ORI formulations have been developed such as nanosuspensions, liposomes, nanogels (Duan et al., 2011; Wang et al., 2017). In previous studies, lots of ORI-loaded nanoparticles have been developed to enhance ORI tumor-targeting efficiency and antitumor activity, for example ORI-loaded solid lipid nanoparticles (Wang et al., 2014), ORI-loaded poly (ε-caprolactone)- poly (ethylene oxide)-poly (ε-caprolactone) copolymer nanoparticles (Feng et al., 2008), ORI-loaded peptide Arg-Gly-Asp (RGD)-modified poly(D,L-lactic acid) nanoparticles (Xu et al., 2012). Comparing with the above nanoparticles, the as-synthesized PLGA-PEG nanoparticles-based drug delivery system in this work demonstrated higher drug-loading rate and entrapment rate, lower immunogenicity, etc., In particular, the synthetic method is simple and the synthetic conditions are easy to control.

**FIGURE 7:**
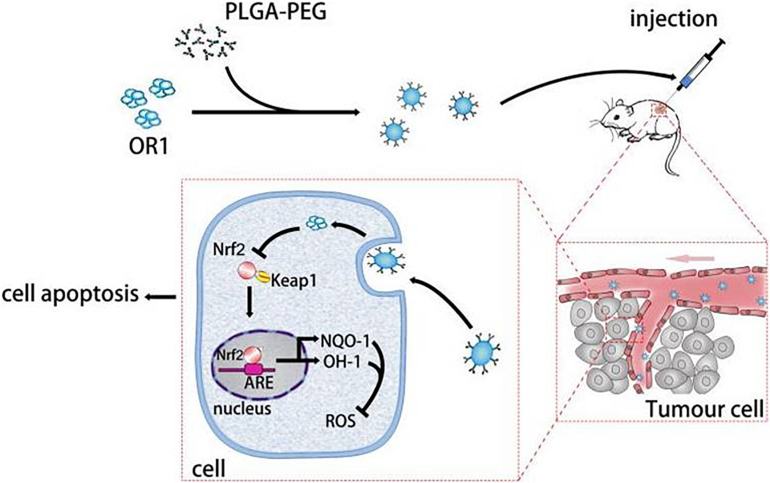
Schematic illustration of the ORI-NPs theranostic nanoplatform.

In this study, we prepared PEG-PLGA NPs as carriers to deliver ORI for the therapy of breast tumor. The ORI-NPs exhibited a slow and sustained release of ORI *in vitro* and *in vivo*. Furthermore, ORI-loaded PEG-PLGA NPs exerted significant anti-tumor effects through promoting cell apoptosis via increased ROS production, which was associated with suppressed HO-1 expression, and Nrf2 nuclear translocation (Figure 7). In addition, ORI-NPs showed prolonged retention in the circulation, and inhibited tumor cell growth, migration, and angiogenesis in tumor xenografts, suggesting that PEG-PLGA based nanomaterials could be used as effective drug delivery systems in cancer therapy.

ROS plays an important role in the signal transduction pathways that regulate cell growth, cell metabolism and redox status, while overproduction of ROS can destroy lipids, proteins and DNA, leading to cell death (Sharma et al., 2012; Wheaton et al., 2014). The increase in ROS and a consequent loss of mitochondrial membrane potential (shown in Figure 4) are typical phenomena during mitochondria-dependent apoptosis. This study demonstrated that the elevated ROS production by ORI-NPs contributed to the cell death, which could be reversed by the ROS scavenger, suggesting the involvement of ROS production. The data also demonstrated that ORI-NPs-dependent increases in cell death were associated with Nrf2 signal pathway, i.e., ORI-NPs inhibited the nuclear expression of Nrf2 and raised the cytoplasmic expression of Nrf2 (Figure 4).

In pharmacokinetics assay, we found that ORI was completely removed from the circulation within 1 h, while the ORI-NPs exhibited a significantly prolonged blood clearance (Figure 6A). Angiogenesis not only contribute to tumor growth by supplying sufficient nutrients and oxygen, but also leads to tumor metastasis. In mouse xenograft model, we found that application of ORI-NPs significantly blocked the neovascularization of tumor xenografts, implying that ORI-NPs possess the anti-angiogenesis activity.

In summary, we developed PEG-PLGA nanoparticles as a drug delivery system to improve the anti-cancer efficiency of ORI. The NPs facilitated the cellular uptake and improved the cytotoxicity of ORI in breast tumor cells. In addition, the NPs exhibited a long circulating property *in vivo* and improved anti-breast cancer efficacy of ORI in mice bearing MCF-7 cell xenografts mainly through ROS-related Nrf2 signal pathway. Collectively, all these data indicate that ORI could be as a potential anti-cancer candidate through the regulation of ROS-related Nrf2 signal, and also confirm the ability of PEG-PLGA NPs to carry drugs and display long-term effects to tumors.

## Data Availability Statement

The original contributions presented in the study are included in the article/supplementary material, further inquiries can be directed to the corresponding author/s.

## Ethics Statement

All experimental protocols and procedures were approved by the Animal Ethics Committee of Guangdong Province, China.

## Author Contributions

XL and HJ proposed and supervised the project. YZ, WX, and WP performed the experiments. HJ and YZ wrote the manuscript. CE polished and revised the manuscript. All authors have given approval to the final version of the manuscript.

## Conflict of Interest

The authors declare that the research was conducted in the absence of any commercial or financial relationships that could be construed as a potential conflict of interest.
